# Modularisation of published and novel models toward a complex KIR2DL4 pathway in pbNK cell

**DOI:** 10.1016/j.mex.2022.101760

**Published:** 2022-06-16

**Authors:** Nurul Izza Ismail, Ana Masara Ahmad Mokhtar

**Affiliations:** a School of Biological Sciences, Universiti Sains Malaysia, 11800 USM, Penang, Malaysia; b School of Industrial Technology, Universiti Sains Malaysia, 11800 USM, Penang, Malaysia

**Keywords:** Mathematical model, Modularisation, Intracellular signalling pathway

## Abstract

KIR2DL4 is an interesting receptor expressed on the peripheral blood natural killer (pbNK) cell as it can be either activating or inhibitory depending on the amino acid residues in the domain. This model uses mathematical modelling to investigate the downstream effects of natural killer cells’ activation (KIR2DL4) receptor after stimulation by key ligand (HLA-G) on pbNK cells. Development of this large pathway is based on a comprehensive qualitative description of pbNKs’ intracellular signalling pathways leading to chemokine and cytotoxin secretion, obtained from the KEGG database (https://www.genome.jp/pathway/hsa04650). From this qualitative description we built a quantitative model for the pathway, reusing existing curated models where possible and implementing new models as needed. This model employs a composite approach for generating modular models. The approach allows for the construction of large-scale complex model by combining component of sub-models that can be modified individually. This large pathway consists of two published sub-models; the Ca^2+^ model and the NFAT model, and a newly built FCεRIγ sub-model. The full pathway was fitted to published dataset and fitted well to one of two secreted cytokines. The model can be used to predict the production of IFNγ and TNFα cytokines.

• Development of pathway and mathematical model
• Reusing existing curated models and implementing new models
• Model optimization and analysis

## Graphical abstract

**Figure:**
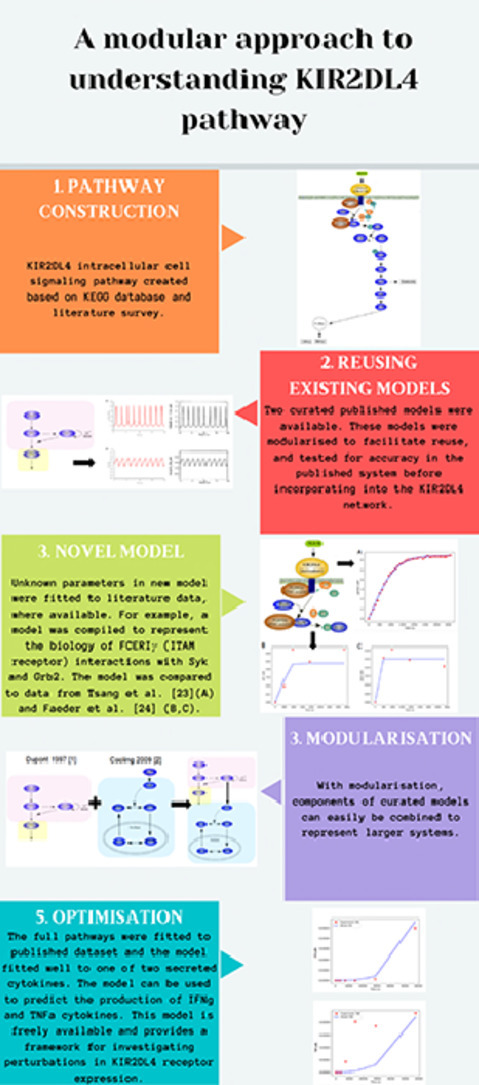

## Specifications table

**Table:** 

Subject Area:	Bioinformatics
More specific subject area:	*Mathematical model*
Method name:	*Modular approach*
Name and reference of original method:	*N.A.*
Resource availability:	*CellML (*https://www.cellml.org/*)*

## Method details

**Fig. 1:**
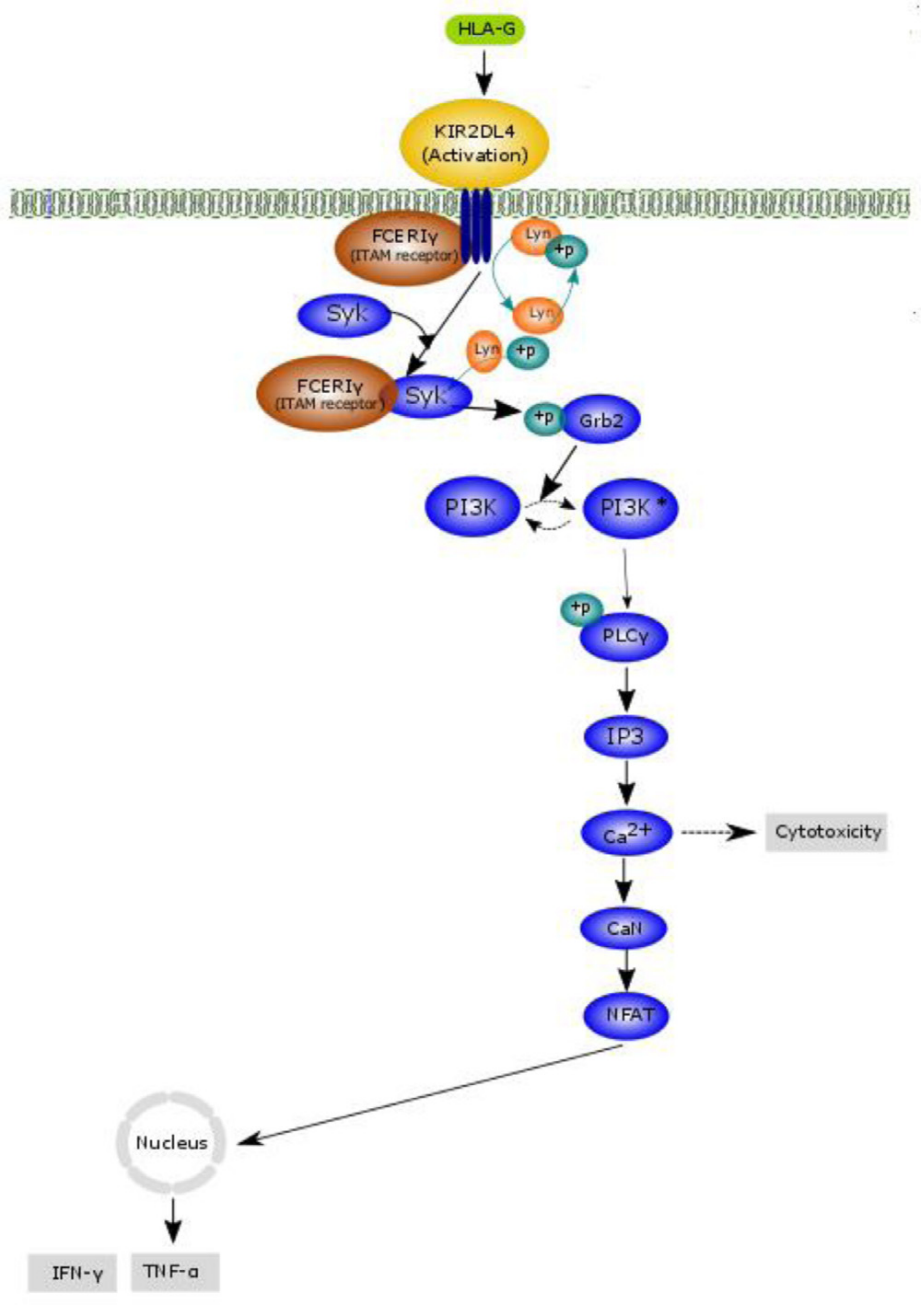
Schematic representation of TNFα and IFNγ secretions induced by HLA-G signalling pathway. Soluble HLA-G has been known to be a ligand for KIR2DL4. Soluble HLA-G originates from cell surface-bound HLA-G. Metalloproteinase (a protease enzyme) is responsible for the cleavage/release of HLA-G from the surface. The experimental setting used to fit the data also used soluble HLA-G to stimulate KIR2DL4. In our model, we assumed that the HLA-G that binds to KIR2DL4 is soluble HLA-G. The transient passage of endocytosed KIR2DL4 receptor at the cell surface occurs when an NK cell is activated by IL-2 is sufficient to capture soluble HLA-G and transport it to the endosomes. The endocytosed HLA-G/KIR2DL4 complex recruits FCεRIγ and aggregate FCεRIγ. FCεRIγ activation is known to induce phosphoinositide 3-kinase (PI3K) [5,6]. PI3K-mediated production of phosphatidylinositol 3,4,5-triphosphate (PtdIns(3,4,5)P3) allosterically enhances PLC activity downstream. This early signalling pathway then activates the NFAT futile cycle and initiates the regulation of IFNγ and TNFα secretion in NK cells.

In this study, we developed a model to predict the production of interferon gamma (IFNγ) and tomur necrosis factor alpha (TNFα) cytokines induced by the binding of human leucocyte antigen (HLA)-G to endocytosed killer cell immunoglobulin-like receptor 2DL4 (KIR2DL4) receptor as shown in Figure 1. The KIR2DL4 receptor is an endocytosed receptor. The transient passage of endocytosed KIR2DL4 receptor at the cell surface occurs when an natural killer (NK) cell is activated by interleukin-2 (IL-2). The transient passage of KIR2DL4 at the cell surface is sufficient to capture soluble HLA-G and transport it to the endosomes. The endocytosed HLA-G/KIR2DL4 complex recruits FC epsilon RI gamma (FCεRIγ) and aggregates the FCεRIγ. Activation of FCεRIγ by Lyn-catalysed phosphorylation then recruits Syk. Syk binding to FCεRIγ is activated via phosphorylation by Lyn. Phosphorylated Syk then phosphorylates GRB2-associated binding protein 2 (GAB2), which leads to activation of the phosphoinositide 3-kinase (PI3K) signalling cascade. One pathway activated by HLA-G through PI3K is the PLCγ-IP3-Ca-CaN-NFAT cascade. Ligation of FCεRIγ stimulates expression of many cytokines via a Ca-dependent mechanism. The PLCγ-IP3-Ca-CaN-NFAT cascade also can be activated by active Syk/ZAP70 via LAT. PI3K and linker of activated T cells (LAT) activate phospholipase C gamma (PLCγ). The role of PLCγ is to control the concentration of calcium (Ca^2+^) in the cell [1,2] through IP3. PLCγ catalyses the conversion of IP3 from PIP2. IP3 causes the release of Ca^2+^ from the intracellular endoplasmic reticulum. Ca^2+^ binds to calcineurin (CaN), and the complex then dephosphorylates the nuclear factor in an activated T-cell (NFAT). The migration of NFAT into the cell nucleus starts the transcription of cytokines TNFα and IFNγ [3,4].

**Fig. 2:**
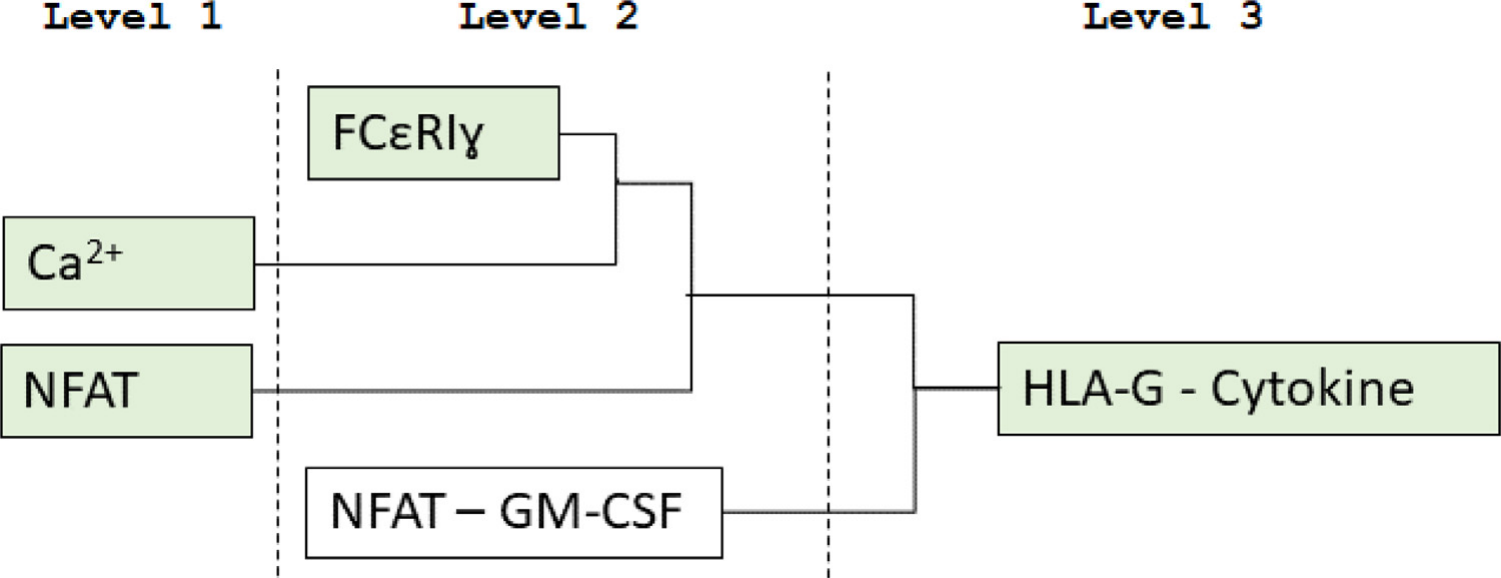
A phylogenetic tree showing the evolutionary relationships among existing and new sub-models of the pathway. We consider three distinct levels of models. Level 1 modules are created from models of components of the signalling pathways that already exist in the literature. Level 2 modules are small connected components of the signalling pathway that determined by the availability of experimental data which allows the parameters of the modules to be define. Level 3 combined all sub-models together to create a whole model. Sub-models highlighted in green boxes mean experimental data exists.

The signalling pathway to be modelled, depicted in Figure 2 consist of many components and reactions. We apply modular design principles into the construction of NK signalling model. This model incorporates two published models including the Ca^2+^ oscillations of Dupont and Erneux (1997) [7] and NFAT cycling of Cooling et al. (2009) [8]. A new sub-model was built to complete the signalling pathway, based on the availability of experimental data. One sub-model was developed in this section and termed FCεRIγ and appear as Level 2 models in Figure 2. With all required sub-models developed, we were then able to bring together these models into a complete HLA-G-KIRDL4 pathway and labelled as Level 3 in Figure 2.

## Materials and Methods

### Model construction

To identify appropriate signalling pathways to derive models at all levels and models that had been previously published that covered components of the system we used online databases as resources for signalling pathway and experimental data. KEGG (https://www.genome.jp/kegg/pathway.html) is the main source for cell signalling pathways. Several model repositories of computational models of biological processes were searched for existing models. The databases searched included PMR (https://models.physiomeproject.org/welcome) and BioModels (https://www.ebi.ac.uk/biomodels-main), where the first is the CellML model repository and the latter is the SBML model repository. The models in those repositories are published in either curated and non-curated form. We also extended the data search into the JWS Model Database (http://jjj.biochem.sun.ac.za), an online modelling database that allows online simulation of models. Models described in the literature were manually curated and enriched with cross-references.

### Re-using components of existing model in CellML

**Fig. 3:**
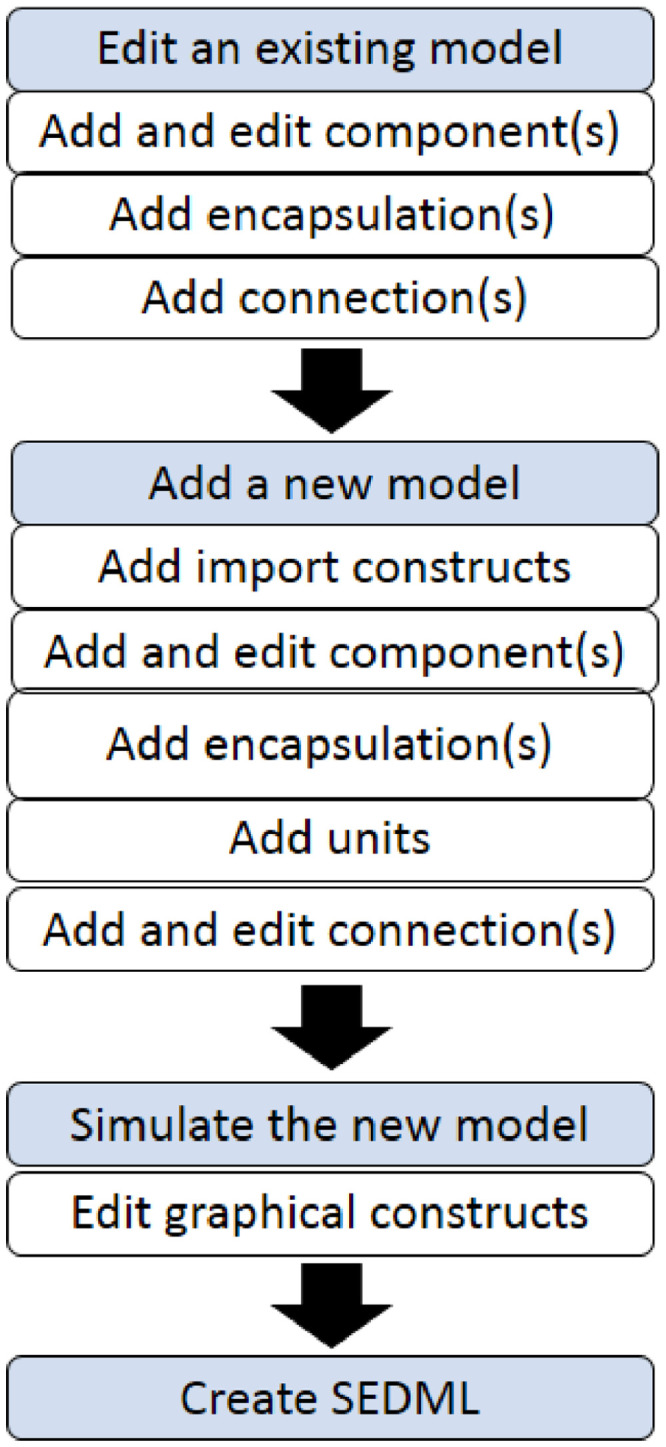
Flow chart of steps in re-using and importing components into a new model. Model components in CellML contain units, variables and equations and can be connected to other components through mapping to allow information to be shared between the components. The CellML encapsulation feature allows users to encapsulate components, either to hide the complexity of a model by creating submodels, or to provide mechanisms for plugging in different implementations of a particular detail of a model.

To re-use an existing model, we first re-simulated the model to ensure the model outputs were the same as published outputs. We then imported component(s) or variable(s) that we intended to re-use in our model. We then encapsulated the components. The steps in re-using and importing components from existing models to a new top-level model are shown in simplified form in a flow chart provided in Figure 3.

**Fig. 4:**
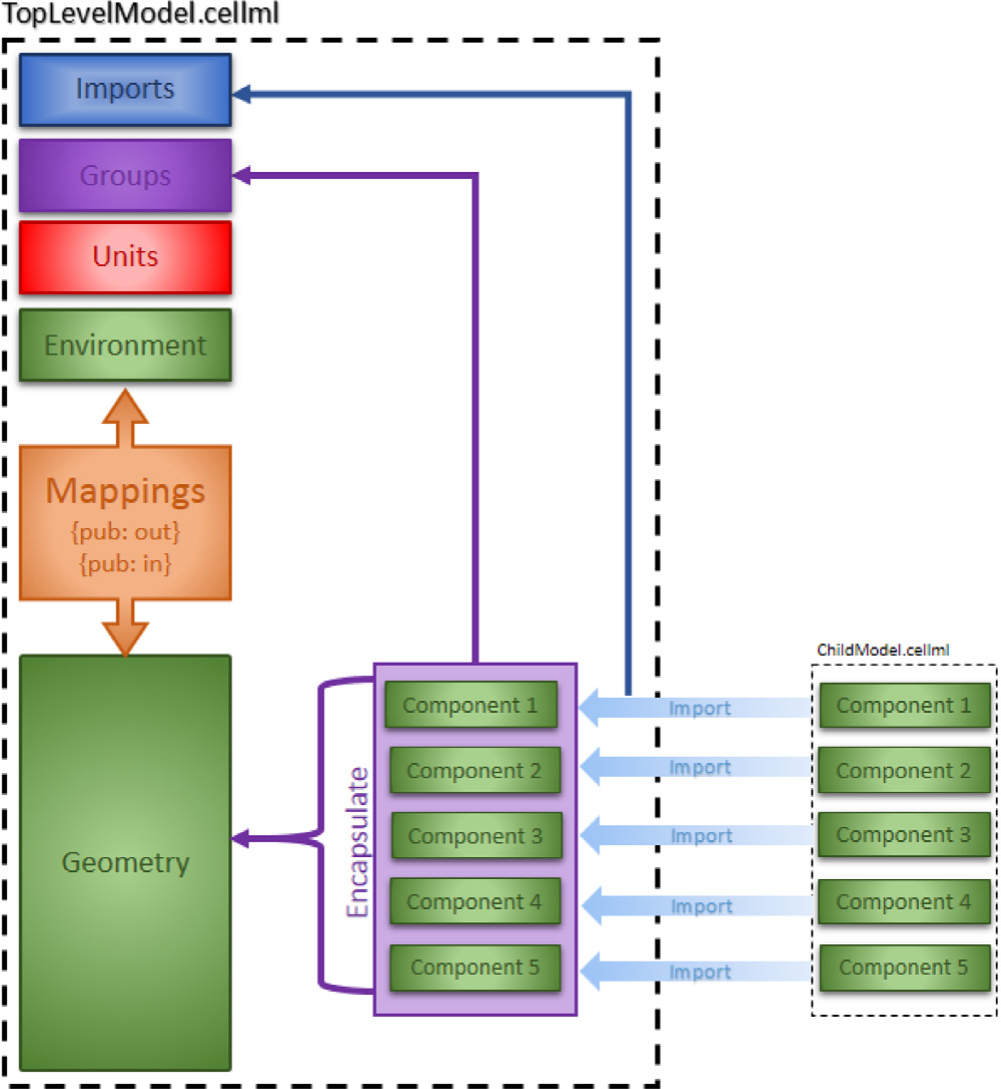
Overall structure of the top level CellML model showing the encapsulation hierarchy, the CellML model imports and the other key parts (units, components, and mappings).

The new model is usually a top-level model, which may contain the CellML sub-model(s) imports, the encapsulation hierarchy, and the units, components, and mappings of the new model. The top-level model and sub-models are linked using public mapping and private-public mapping if encapsulation is used. The CellML model of a top-level model with sub-model(s) imports is provided in Figure 4. We then tested the model to ensure it worked as expected.

### Methods for searching for experimental data

Several methods were used to gain information for the experimental data in this study. Various keywords for data searches were used, ranging from general to specific terms, and combinations of both vague and specific keywords. The data search was done for NK cell signalling experimental output, and for the signalling experimental output for other immune cells including T cells, B cells and macrophages. We extended the data search to other non-immune cell types to ensure we obtained all available data. But we tried to make use of data from immune cells first. The search keywords included single molecule keywords such as HLA, KIR, PI3K, and Vav. We also used combinations including trophoblast-NK cell, receptor-ligand combinations, receptor-cytokine combinations, receptor/ligand-molecule combinations, molecule-molecule combinations, molecule-cytokine combinations.

We also referred to the Protein Data Bank (PDB) (https://www.rcsb.org), a database for three-dimensional structural data for large biological molecules, such as proteins and nucleic acids, for annotated collections of publicly available molecular structures, using relevant data such as molecular weight. Large amounts of additional relevant data are available through databases such as the Gene Expression Atlas (https://www.ebi.ac.uk/gxa/home), which is an open resource database that provides a huge quantity of information about gene and protein expression. The data relevant to signalling pathways that we could access in this database dealt with the relative expression of genes.

Once a model has been developed, enough experimental data is crucial to ensure that the *in silico* simulation is reliable. The data sources can vary from journals to laboratory findings from experiments. Our model incorporated a wide variety of empirical observations ranging from human to non-human primates. Data that we needed included initial conditions of species involved in the pathway and kinetic rates of reactions. We examined literature data including integrative pathway diagrams, quantitative and qualitative details of possible molecular states, interaction, and activities [9]. Appropriate parameter values were chosen from the literature where possible and others were optimised using parameter fitting.

### Initial conditions

In this study, the initial conditions for substrates were derived from literature where possible. The initial conditions depend on the experiment conducted or be fit if unknown. For experimental data that using the same unit, the value can be used directly. In some circumstance, experimental data was generated in different unit to the model, i.e., pg/ml, so the data need to be converted to molarity using a formula as follows

$$\text{Molar}\;\text{concentration},\;\;c_{i}=\frac{P_{i}}{M_{i}}$$

where p is the density of constituent i, and M is the molar mass of constituent *i*.

For substrate without any data, we used the relative initial condition that has been optimised for similar reaction or we made early assumption within the relevant range. Once we optimised the model, we then decide whether we need to increase or decrease the value. The complexes in the system were fixed at 0 μM at time t =0.

### Model calibration (parameter estimation)

Here we address the parameter estimation using least-squares optimisation in Python. The parameter estimation implements the Levenburg-Marquardt gradient method (greedy algorithm) to minimise an objective function [10]. The first step is to define the objective function to minimise. Gradient methods such as Levenburg-Marquardt tend to run into the nearest local minimum. The method is also sensitive to initialisation of parameters to be fit. The algorithm iteratively solves a trust-region sub-problems augmented by a special diagonal quadratic term and with trust-region shape determined by the distance from the bounds and the direction of the gradient. These enhancements help to avoid making steps directly into bounds and efficiently explore the whole space of variables. Traditional gradient-based local optimization methods normally fail to get to a global minimum.

As the least-square method is prone to finding a local minimum rather than a global minimum [11,12], it is important to be reasonably close to the global minimum to get the best fit. To surmount this limitation, we must conduct a sweep of parameter space to find suitable search regions [13]. However, for many of our models, there are several unknown parameters, and so parameter space is too large to practically cover simply by varying parameters one by one. We therefore, aim to randomly sample parameter space with suitable coverage to be able to assure that we can find solutions close to a global minimum [14]. We use a sampling method to create a random sample of parameters within identified boundaries [15,^16^,17]. It allows users to determine the sampling size where the big sample size gives more chance for a better fit. It then runs the model lots of times using sampled parameter space and ranks the parameter set by the objective function. The parameter samples were generated using the Saltelli sample function stored in Python modules [15,^16^,17] as following

Nx(2D+2)

where N is the scaling factor of samples to generate (the argument we supplied) and D is the number of model parameters. Parameter sweep generates samples within the specified parameter space.

We then generally loop over each sample input and evaluate the model. The parameter fitting was conducted via the optimisation of objective function. The objective function was defined as the sum of the squared differences between predicted and experimental values. The equation is defined as

$$S=\sum_{i=1}^{n}{\left(x_{i}-\bar{x}\right)}^{2}$$

where S is the sum of the squared differences between experimental and predicted values.

The unknown parameters may cover orders of magnitude, so we need to cover solutions, equally and fairly over a large parameter sweep. For this reason, the fitting assumes exponents for parameter bounds. For example, a lower bound of -3 and an upper bound of 2 will sweep parameters between 10^−3^ and 10^2^. In this study, we used the default tolerance for termination by the change of the cost function and termination by the change of the independent variables. Default value set by the leastsq for both tolerances is 1e-8. The lower and upper bounds on independent variables are also set for each parameter.

### Model analysis (sensitivity analysis)

The drawback of developing a big model in a small compartment is that the non-sensitive parameter from the small sub-model can be inherited to the bigger sub-model. Sensitivity analysis is one of the methods to access uncertainty in model parameter and can be used to identify parameter that need to be quantified experimentally. Sensitivity analysis is the study of how the inputs of a given model can change solution or output of the model, particularly of how the different level in the input of a model can be qualitatively or quantitatively apportioned to different outputs of a model [14,^18^,19].

To perform the analysis to NK cell model, each sub-model was analysed by looking at how sensitive is the model to each involved parameter. For small sub-models we use the one parameter at a time approach. That is, we change one parameter at a time and keep the other parameters fixed and investigate how that parameter impacts the objective function error. We initialised the model with the best fit values for each parameter and ran it multiple times over the parameter range. We varied each parameter from its best fit value, over the parameter range we assumed it to take. The analysis is repeated for all the parameters. We vary parameters over the feasible parameter range to check how much they impact solutions. We achieve this by using the inbuilt Saltelli function in the python SALib library [14].

Error defines the differences between points in model solution and experimental solution. In this study, we used least-squares method which calculated the sum of the squared points of model from data. We plot a graph of the error (shown on the y-axis) versus parameter range (shown on the y-axis). Dips in the error correspond to local minima in the parameter space. If a model is sensitive to a parameter, it remained fixed in subsequent simulations. At times the minimum was right at the edge of the parameter space. This parameter showed distinct changes in model behaviour on one side of the minimum can be varied during fitting to another set of data in the range around the minimum in which the line is flat. This means this parameter value needs to be around the ’minimum’. A flat line showed that the model is not sensitive to those parameters. The latter two types of parameters can be varied in fitting to subsequent data sets.

### Physiologically Feasible Parameter Combinations That Can Replicate Cell Function

While the parameter fitting was looking for a ’best fit’ solution, which is set of parameters that provides the minimum error, this best fit may not be the only feasible model parameterisation that provides a close similarity to the experimental data. Alternate sets but fundamentally different solutions may be existed. Thus, we search for solutions within the parameter space that predicts concentrations of IFNγ and TNFα that varies within twice the value of the mean. We implemented a custom python script to sample the parameter space formed by the 23 parameters that can be fitted. Here, we implemented again Saltelli sampling function [16].

By selecting parameters in this way we are able to conduct a global analysis of model behavior, in that we vary all parameters simultaneously over the whole parameter space. In a formal global (e.g. Sobol) sensitivity analysis [20] the value of N must be sufficiently high to allow for statistical interpretation of the variability in model outputs. Saltelli sampling is an extension of the Sobol sequence, which is a quasi-random sequence that aims to provide a relatively uniform coverage of sampling space [17,21].

### Modelling intracellular signalling of HLA-G-KIR2DL4 pathway

a. Re-using and implementing models from Physiome Model Repository (level 1 models)
 i. The Ca^2+^ model. Effects of inositol 1, 4, 5-trisphosphate 3-kinase and 5-phosphatase activities on Ca^2+^ oscillations

**Fig. 5:**
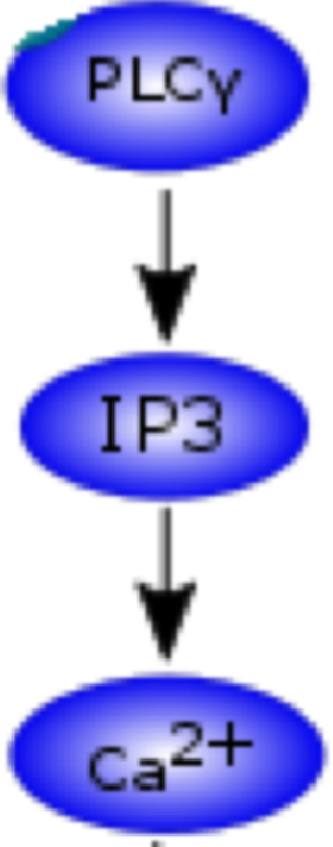
Pathway of interest from Dupont & Erneux model [7]. Activation of PLC synthesizes IP3, which then induces the release of Ca^2+^ from internal stores in the cell. IP3 is a protein known to stimulate the recruitment of Ca^2+^ from cellular stores. IP3 binds to IP3 receptors located on the membranes of intracellular stores of Ca^2+^ and stimulates the release of Ca^2+^ into the cytosol. Ca^2+^ activation happens rapidly in the system. At the same time, ATPases pump Ca^2+^ back from the cytosol to the stores.

This section outlines the model labelled as Ca^2+^ sub-model in Figure 2. Dupont and Erneux simulated a model that captures the oscillation of Ca^2+^ in a cell in response to upstream signalling (Figure 5) [7]. We implemented the Ca^2+^ oscillations phenomenon from Dupont & Erneux, 1997 in our model (https://github.com/Nurulizza/HLAG_to_cytokine/tree/master/dupont_Ca).

This model describes the link between activation of phospholipase C (Plc) and Ca^2+^ dynamics in the cell. IP3 activates the release of Ca^2+^ from internal stores via IP3 receptors. The model simulation showed that IP3 and IP4 oscillations are passively controlled by the cytosolic Ca^2+^ oscillations. The oscillations are caused by feedback regulation of cytosolic Ca^2+^ on the IP3 receptor.

The concentration of cytosolic Ca^2+^ is given by the following equation

$$\frac{d\left[C_{cyto}\right]}{dt}=k_{1}\left(b+I_{ra}\right)\left(Ca_{tot}-C_{cyto}\left(\alpha +1\right)\right)-V_{MP}\frac{C_{cyto}^{n_{p}}}{K_{p}^{n_{p}}+C_{cyto}^{n_{p}}}$$

where C_cyto_ and Ca_tot_ are the concentration of cytosolic Ca^2+^ and total Ca^2+^. k_1_ describes the kinetic rate of Ca^2+^ from the stores into the cytosol, I_ra_ is the fraction of active channels. α represents the ratio between the volumes of the intracellular stores and the cytosol and b accounts for a basal efflux from the stores into the cytosol. Translocation of Ca^2+^ back to stores is controlled by cytosolic Ca^2+^, described using the Hill approximation where VMP, Kp and np describe the maximum velocity, the constant for half maximum activity, and the Hill coefficient, respectively.

In this model, Dupont and Erneux observed the typical behaviour of cytosolic Ca^2+^ oscillations and the influence of Ca^2+^ oscillation on the oscillation of IP3 and IP4 concentrations. This happens because each Ca^2+^ spike activates IP3 3-kinase, which further stimulates the change of IP3 into IP4. The amplitudes of IP3 and IP4 are determined by the stimulation level and by the maximum velocity and threshold constant of the IP3 3-kinase. The model was verified against the paper.

 ii. The NFAT model. Sensitivity of NFAT cycling to cytosolic Ca^2+^ concentration.

This section describes the model labelled as ‘NFAT’ in Figure 2. The nuclear factor of activated T-cell (NFAT) transcription factors is stimulated by Ca^2+^ signals. In our model, the activation of NFAT is also stimulated by Ca^2+^. In NK cell, the activation and translocation of NFAT into the nucleus stimulate the production of cytokines. In 2009, Cooling et al. [8] simulated NFAT activation and translocation to the cell nucleus to regulate gene transcription. They replicated important regulation of Ca^2+^ oscillations and CaN to downstream NFAT cycling. We implemented Cooling et al.’s 2009 model [8] in our model, which includes the same pathway (https://github.com/Nurulizza/HLAG_to_cytokine/tree/master/cooling_NFAT).

**Fig. 6:**
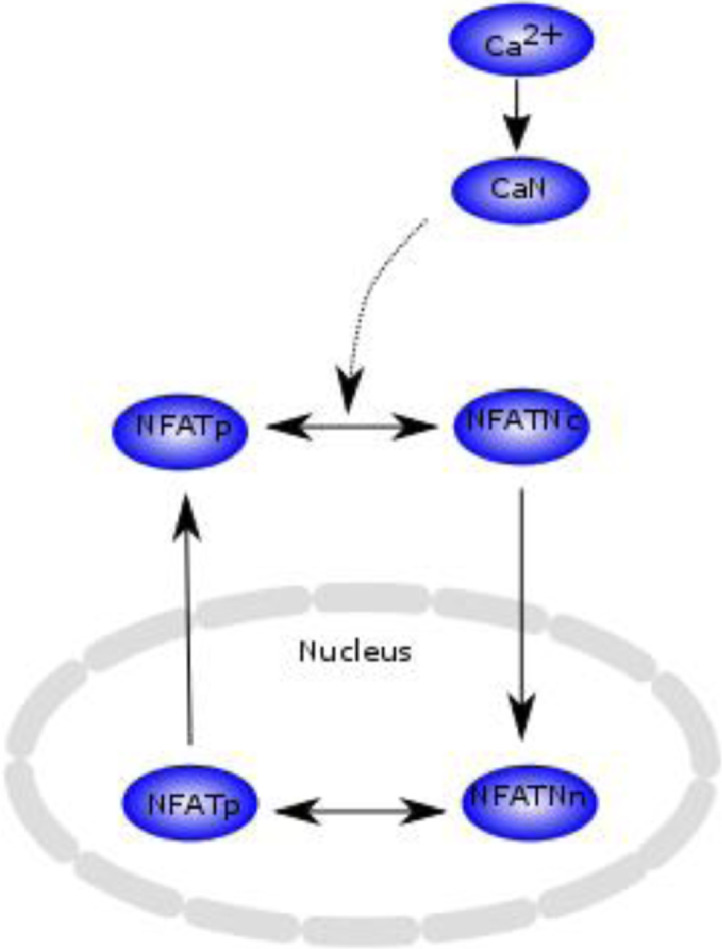
Pathway of interest from the Cooling et al. model [8]. To illustrate the details of the reaction, we show the translocation reaction in this diagram. NFAT is stimulated by calcium signals. The activation and translocation of NFAT into the nucleus stimulate the production of cyokines. We replicated important regulation of calcium Ca^2+^ oscillations and calcineurin (CaN) to downstream NFAT cycling.

The IP3-Ca^2+^-CaN pathway plays an important role in the stimulation of transcription factor NFAT [8]. Signals from upstream Ca^2+^-CaN dephosphorylate NFAT in the cytoplasm. During dephosphorylation, NFAT is translocated into the nucleus. In the NFAT cycling model built by Cooling, Ca^2+^ activates calmodulin-calcineurin complex (calmodulin is not shown in Figure 6 as it forms complex with CaN), which further binds to NFAT and dephosphorylates it in the cytosol. The dephosphorylated NFAT translocates to the nucleus. NFAT may also be rephosphorylated and translocated back to the cytosol.

In this model, CaN is activated by Ca^2+^. Activated CaN then dephosphorylates NFAT. The dephosphorylation of NFAT allows the translocation of NFAT to the cell nucleus. In this model, we assumed that NFAT can be rephosphorylated by a number of kinases. Once NFAT is rephosphorylated, it is exported back to the cytoplasm.

The model has six unknown parameters, which were fitted to data from baby hamster kidney (BHK) cells. The model implements an approach defined by Tomida et al. [22] to investigate Ca^2+^ effects in BHK cells. The outputs from the model show that an oscillating Ca^2+^ signal is more efficient than a constant Ca^2+^ signal. The size of the Ca^2+^ signal determines the NFAT cycling rate. The implementation of this model allows us to replicate Ca^2+^ mobilization in NK cells. The model also simulated the dephosphorylation of NFAT in the cytosol. From their model, they also found out that the dephosphorylated NFAT in the cytosol is buffered before declining. Nuclear NFAT was observed to rise steadily, as seen in the experimental data. The model was also able to replicate the effect of over expression of CaN on the NFAT cycle. The model showed a normal NFAT cycle with over expressed CaN at the same time that Ca^2+^ is set at a constant level. In our cell signalling, the translocation of NFAT into the cell nucleus leads to secretion of cytotokines.

To simplify the model, as in the original publication [8], NFAT phosphorylation and dephosphorylation reactions are modelled as single steps, although in nature there is more than one phosphorylation site involved. The model also assumed a single step for NFAT translocation. The NFAT translocations into and from the nucleus do not have back reactions. This process is described using a futile cycle in the system.

With these assumptions, the phosphorylation and dephosphorylation of NFAT, together with translocations of NFAT into and from the nucleus, are described using mass action kinetics. The phosphorylation and dephosphorylation are described as

$$NFATp_{c}+N_{active}↔NFATN_{c},$$

$$J_{1}=\;k_{f1}\;x\;NFATp_{c}\;x\;N_{tot}\;x\;act_{N\;}x\;-k_{r1}\;x\;NFATN_{c}\;x\;\left(1-act_{N}\right),$$

where N_active_ is the amount of activated CaN, determined by the total CaN, N_tot_ and fraction of activated CaN, Act_N_. Thus, the translocations of NFAT into and from the nucleus are described as

$$NFATp_{n}\to NFATNp_{c},$$

$$J_{2}=NFATN_{c}\;x\;k_{f2},$$

$$NFATN_{c}\to NFATN_{n},$$

$$J_{4}=NFATp_{n}\;x\;k_{f4}.$$

Parameter fitting was performed against the data of Tomida et al. [22], by Cooling et al. [8] and the model was verified against the paper.

b. Implementing novel pathway models (level 2 models)

**Fig. 7:**
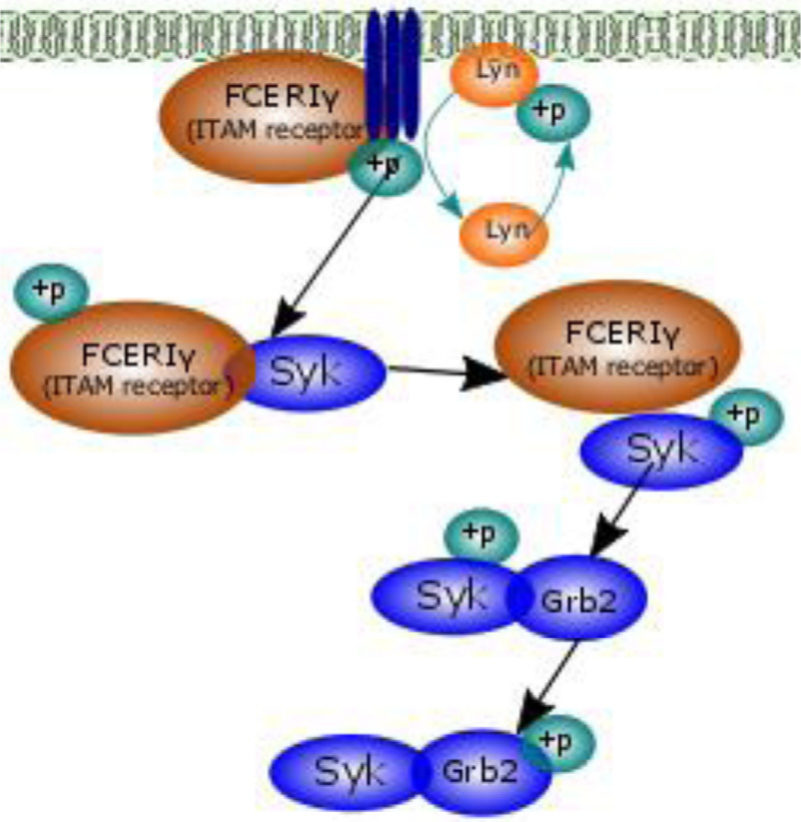
Schematic diagram of the FCεRIγ signalling pathways. Two sets of experimental data were available in the literature for this pathway; Tsang et al. (2008) [23] and Faeder et al. (2003) [24]. In this model, we simulated the phosphorylation of Grb2 and Syk downstream to the activation of FCεRIγ.

A new sub-model that was built to complete the whole signalling pathway, based on the availability of experimental data. One sub-model was developed in this section and termed FCεRIγ and appear as Level 2 models in Figure 2. The repository associated with this model can be found at https://github.com/Nurulizza/HLAG_to_cytokine/blob/master/FCepsilonRI.cellml. Two sets of experimental data were available in the literature for this pathway. One was published by Tsang et al. [23] and the other by Faeder et al. [24]. This model was built based on data from both literatures. Previously Faeder et al. developed a model of early signalling of FCεRIγ [23], however, this model was focussed on the receptor itself and contained a greater level of complexity than was required in our model. In this sub-model we aimed to build a simple model of signalling of FCεRIγ that still represented biological understanding and fitted the experimental data (Figure 7). FCεRIγ exists in tetrameric form, which has an α-chain, a β-chain and two disulfide-linked γ-chain [24]. The α subunit can be bound to ligand or unbound, a β-chain has four possible states of phosphorylation and two disulfide-linked γ-chain has six states of phosphorylations. In total, there are 300 possible states of receptor subunits for FCεRIγ [24]. To avoid complexity, we assumed that FCεRIγ is in an aggregated state and ready for binding with other reactants. The activated FCεRIγ receptor is transphosphorylated by pLyn, available at the cell surface. This followed by the recruitment of Syk to the membrane surface which is subsequently transautophosphorylated by phosphorylated FCεRIγ. Inactive Grb2 then binds to phosphorylated Syk, and becomes phosphorylated.

Syk is a tyrosine kinase that is important in bridging receptor ligation and down- stream signalling such as Ca^2+^ and MAPK. Once the cell receptor binds with the ligand, FCεRIγ (ITAM receptor) is recruited and transphosphorylated by Lyn. The phosphorylated ITAM then recruits protein tyrosine kinase (Syk). Previous studies have shown that ITAM phosphorylation increases Syk activity and modulates Syk potency [23]. It must be noted that concentration above 10 µM ITAM can inhibit Syk activity [23]. Phosphorylated Syk then phosphorylates Grb2 (growth-factor-receptor-bound protein 2). Phosphorylation of Grb2 leads to the activation of PI3K downstream signalling. To match the available experimental data, we assumed that the Lyn kinase is rephosphorylated for recruitment of additional pLyn into the system to ensure that the system has enough supply of phosphorylated Lyn. The equation associated with this reaction is

$$Lyn+Pi\;\overset{k_{3}}{\to }\;pLyn$$

where Pi denoted a phosphate, k denotes a forward reaction and pLyn denotes a phosphorylated Lyn. The flux associated with this reaction is

$$J3=k_{3}\left[Pi\right]\left[Lyn\right],$$

where J denotes a flux, k denotes kinetic constant and Pi denotes a phosphate.

Experimental detail on Syk activity in B cells is presented in the study by Tsang et al. [23] which captures the molecular mechanism of Syk activation in vitro. The studies explore Syk activity under various conditions: activities of phosphorylated and non-phosphorylated Syk; activation of Syk by different receptors; and whether or not Syk is an OR-gate type of molecular switch. Syk activity is required for more than one hour to induce activation of downstream reactions, so the experiment was run for 3600 s. For that reason, the model simulation time was set for 3600 s. For the FCεRIγ model, we had 10 unknown parameters to fit to this data.

In one of the observations, Tsang looked at the activity of Syk when bound to FCεRIγ [23]. The experimental data we fitted the model to were for a temporal Grb2 phosphorylation by dephosphorylated Syk with 1 µM FCεRIγ. The Syk concentration used in the experiment was 0.005 µM. Experimental data points were digitised from Supplemental Figure 2 in Tsang et al. [23]. The observed experimental data points were from spectrophotometrical measurements of phosphorylation in a single representative experiment [23]. The binding of Syk to the receptor eliminated the lag phase in Grb2 phosphorylation.

The second data set was adapted from Faeder et al. [24]. In their simulation, Faeder et al. looked at the pathway in rat basophilic leukemia (RBL) cells. The experimental environment was maintained at a temperature of 27°C. The cell density and cell volume were assumed to be 1×10^6^ cells/ml and 1.4×10^9^ ml. The observed experimental data points were from densitometric measurements of phosphorylation in a single representative experiment [25]. Experimental data points were digitised from Figures 3(b) and 3(d) in Faeder et al. [24].

Parameter estimation was performed with each unknown kinetic rate constant allowed to vary in a feasible range that is between 1×10^−3^ and 1×10^2^ [9]. However, we expand the range when we see the optimisation error potentially decreasing with larger boundary. The settings for this work are described in Table 1. We estimated the concentration of Grb2 and pLyn at 6.47 µM and 6.5 µM. The phosphorylated (except Lyn) and complex reactants are assumed to be 0 at time 0 s. The number of sample used for the fitting is 500 which generate 12000 samples.

**Table 1:** Initial conditions for the FCεRIγ signalling sub-model. We adapted Syk and FCεRIγ concentrations from Tsang et al. [23], whilst the concentrations of Grb2 and pLyn at the start of the simulation were estimated.

Parameter	Value	Units	Source
*FCε*	1	*µ*M	[23]
Syk	0.005	*µ*M	[23]
Grb2	6.47	*µ*M	Estimation
pLyn	6.5	*µ*M	Estimation

Tsang and Faeder used different experimental protocols so the initial conditions in our model representing these protocols differed [23,24]. To fit the model to data from Faeder, some initial conditions were derived from the experimental protocol reported, and others were estimated during model fitting [24]. We fitted concentration of FCεRIγ is 0.0474 µM, the concentration of Syk is 0.025 µM, and the concentration of pLyn is 0.0474 µM. The list of initial conditions is listed in Table 2.

c. Modularisation of sub-models toward a complete HLA-G-KIRDL4 pathway

**Table 2:** Initial conditions for the FCεRIγ signalling sub-model. Estimation 1 initial conditions were derived from Faeder et al. [24] and Estimation 2 initial conditions were estimated during model fitting.

Parameter	Estimation 1	Source	Estimation 2	Units
FCε	0.474	[24]	0.0474	µM
pLyn	0.0332	[24]	0.0474	µM
Syk	0.432	[24]	0.025	µM
Grb2	nil	nil	0.01	µM

This section introduces the model labelled HLA-G cytokines in Figure 2. The repository associated with this model can be found at https://github.com/Nurulizza/HLAG_to_cytokine. Experimental studies by Rajagopalan et al. [26] observed the activation of KIR2DL4 by soluble HLA-G. Soluble HLA-G has been known to be a ligand for 2DL4. Soluble HLA-G originates from cell surface-bound HLA-G. Metalloproteinase (a protease enzyme) is responsible for the cleavage/release of HLA-G from the surface. The experimental setting used to fit the data also used soluble HLA-G to stimulate KIR2DL4. In our model, we assumed that the HLA-G that binds to KIR2DL4 is soluble HLA-G.

The transient passage of endocytosed KIR2DL4 receptor at the cell surface occurs when an NK cell is activated by IL-2 is sufficient to capture soluble HLA-G and transport it to the endosomes. The endocytosed HLA-G/KIR2DL4 complex recruits FCεRIγ and aggregate FCεRIγ. FCεRIγ activation is known to induce phosphoinositide 3-kinase (PI3K) [5,6]. PI3K-mediated production of phosphatidylinositol 3,4,5-triphosphate (PtdIns(3,4,5)P3) allosterically enhances PLCγ activity downstream. This early signalling pathway then activates the NFAT futile cycle and initiates the regulation of IFNγ and TNFα secretion in NK cells.

Our model replicates the reaction of NK cells stimulated by KIR2DL4-specific antibody. However, stimulation by the natural ligand, HLA-G, showed a quantitatively similar cytokine response. For most of the secreted cytokines, natural HLA-G induced at least 50% of the amount induced by anti KIR2DL4 mAb [26]. In the experiment, the secretion of TNFα was detected within the first 2 hours and up-regulated more than 2-fold. The secretion of IFNγ was upregulated 1.5-fold after 8 hours of receptor activation.

This large pathway is integrated by three level 1 and level 2 sub-models. Two of the sub-models are published sub-models: the Ca^2+^ model (labelled Ca^2+^ in Figure 2) [7] and the NFAT model (labelled NFAT in Figure 2) [8]. This pathway also includes the FCεRIγ model (labelled FCεRIγ in Figure 2).

Two short reactions needed to be added to the model to connect components of the system. These are a representation of the HLA-G activation and the NFAT cytokines. The first reaction describes the activation of KIR2DL4 by HLA-G. The second reaction describes the secretion of IFNγ and TNFα through activation of NFAT downstream in this pathway.

The reactions that correspond to this portion of the signalling pathway are

hG+2DL4

$$\mathrm{hG}+2\mathrm{DL}4\;\underset{k_{-10}}{\overset{k_{10}}{↔}}\;\mathrm{hG}2\mathrm{DL}4$$

$$\mathrm{hG}2\mathrm{DL}4\;+\;\mathrm{FC}\underset{k_{-1}}{\overset{k_{1}}{↔}}\;\mathrm{hG}2\mathrm{DL}4\mathrm{FC}$$

The fluxes associated with these reactions are

$$J10=k_{10}\left[hG\right]\left[2DL4\right]-k_{-10}\left[hG2DL4\right]$$

$$J1=k_{1}\left[hG2DL4\right]\left[FC\right]-k_{-1}\left[hG2DL4FC\right].$$

Cytokine produces by a cell will be used for cell regulation. Although the model only captures the production of cytokine inside the cell, we added cytokines released from the cell that is applicable to all cytokines. We, therefore, added reactions for cytokine release for both TNFα and IFNγ.

The reactions associated with the reactions are

$$TNF\alpha \overset{k_{31}}{\to }TNF\alpha \;\text{released},$$

$$IFN\gamma \overset{k_{32}}{\to }IFN\gamma \;\text{released}.$$

Parameter fitting was performed using experimental data by Rajagopalan et al. [26]. Each unknown kinetic rate constant varies differently, and the boundaries used for the fitting process are listed in Table 3. At this point, we fixed the initial conditions and kinetic rate constants estimated in individual published sub-models. However, we fitted parameters that were not sensitive to FCεRIγ model.

**Table 3:** Parameters to fit to the data in Rajagopalan et al. [26] and the boundaries condition. Here, we fitted 23 kinetic parameters including 5 parameters that were not sensitive to FCεRIγ model. The boundaries used for the fitting vary depending on sensitivity analysis of kinetic rates of the sub-models.

Parameter	Lower bound	Upper bound
hGactivating/k_f10_	10^−3^	10^2^
hGactivating/k_r10_	10^−3^	10^2^
FCepsilonRI/k_f1_	10^−3^	10^1^
FCepsilonRI/k_f5_	10^−3^	10^0^
FCepsilonRI/k_r1_	10^−3^	10^3^
FCepsilonRI/k_r4_	10^−3^	10^0^
FCepsilonRI/k_r6_	10^−3^	10^0^
hG_FC/k_f21_	10^−3^	10^2^
hG_FC/k_r21_	10^−3^	10^2^
PI3K/k_f2_	10^−3^	10^2^
PI3K/k_r2_	10^−3^	10^2^
PI3K/k_f3_	10^−3^	10^2^
Dupont_NFAT/k_f_	10^−3^	10^2^
NFAT_cytokine/k_f4_	10^−5^	10^5^
NFAT_cytokine/k_f5_	10^−1^	10^3^
NFAT_cytokine/kf_31_	10^−3^	10^2^
NFAT_cytokine/kf_32_	10^−10^	10^−6^
NFAT Cycling equation/kf_21_	10^−3^	10^2^
NFAT Cycling equation/kf_22_	10^−3^	10^2^
NFAT Cycling equation/kf_23_	10^−3^	10^2^
NFAT Cycling equation/kf_24_	10^−3^	10^2^
NFAT Cycling equations/k_r21_	10^−3^	10^2^
NFAT Cycling equations/k_r23_	10^−3^	10^2^
hGactivating/k_f10_	10^−3^	10^2^

We applied initial conditions as observed by Rajagopalan et al. [26] for HLA-G and KIR2DL4. The initial conditions for HLA-G and KIR2DL4 were set at 0.098 µM and 0.1052 µM, respectively. The initial conditions for PI3K was set at 0.01 µM. As shown by the reaction equation above, we assumed that the production of IFNγ and TNFα secretion were parallel to NFAT de-phosphorylation in the nucleus. We estimated the initial condition for plc (non-activated plc) was 1.3 µM, consistent with the initial condition for activated plc. The list of initial conditions adopted from the experiment for the HLA-G activation model, NFAT cytokines model and PI3K activation are shown in Table 4.

**Table 4:** HLA-G cytokines model initial conditions. The initial conditions were adopted from Rajagopalan et al. [26] and Hatakeyama et al. [27].

Parameter	Value	Units	Source
KIR2DL4	0.098	*µ*M	[26]
PI3K	0.01	*µ*M	[27]
HLA-G	0.1052	*µ*M	[26]
plc	1.3	*µ*M	Estimation

## Method validation

### Re-using and implementing models from Physiome Model Repository (level 1 models)

#### Ca^2+^ oscillations in Dupont and Erneux, 1997

This section outlines the model labelled as Ca^2+^ sub-model in Figure 2. Dupont and Erneux simulated a model that captures the oscillation of Ca^2+^ in a cell in response to upstream signalling (Figure 3) [7]. The repository associated with this model can be found at https://github.com/Nurulizza/HLAG_to_cytokine/tree/master/dupont_Ca.

**Fig. 8:**
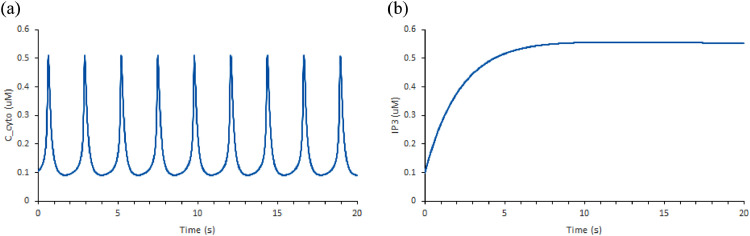
Oscillations for (a) Ca^2+^ and (b) Ins-1,4,5-P_3_ (IP3) metabolism without the production of IP4 for the Dupont and Erneux model. The graphs are simulated in OpenCOR. The simulation retained the oscillation of Ca2+ and the oscillation of IP3 disappeared without the changes of IP3 into IP4. The SED-ML associated with this model is available at https://github.com/Nurulizza/HLAG_to_cytokine/blob/master/dupont_Ca/dupont_erneux_1997_fig2.sedml.

Our pathway does not include the production of IP4 by phosphorylated IP3. To exclude this reaction, we zeroed the V3k reaction in Dupont's model which represents the activity of IP3 3-kinase in catalysing the IP4 production. In this case, when the maximal velocity of 3-kinase is zero, and the activity of 5-phosphatase very much exceeds the activity of 3-kinase, oscillations in IP3 disappear. The behaviour has also been observed in studies of other cell types [28,^29^,30]. The Ca^2+^ and IP3 behaviour in the system without the activity of 3-kinase is illustrated in Figure 8. As expected, we retained the oscillation of Ca^2+^ and the oscillation of IP3 disappeared without the changes of IP3 into IP4.

#### NFAT cycling in Cooling et al., 2009

This section describes the model labelled as NFAT sub-model in Figure 2. Cooling et al. [8] adapted the experimental protocol of Tomida et al. [22], as described previously to model NFAT cycling (Figure 4). The respository associated with this model can be found at https://github.com/Nurulizza/HLAG_to_cytokine/tree/master/cooling_NFAT.

We implemented the original model as published by Cooling et al. in full in our own model since we also included NFAT phosphorylation and the de-phosphorylation futile cycle. The entire model as published by Cooling et al. [8] is used in the NK cell model. To implement the model, we first checked the models in PMR for curation to ensure that the outputs matched the published outputs as described by Cooling et al. [8]. After we satisfied that the model output matched the published outputs, we then incorporated the model into the NK cell signalling pathways.

#### Implementing novel pathway models (level 2 models)

**Fig. 9:**
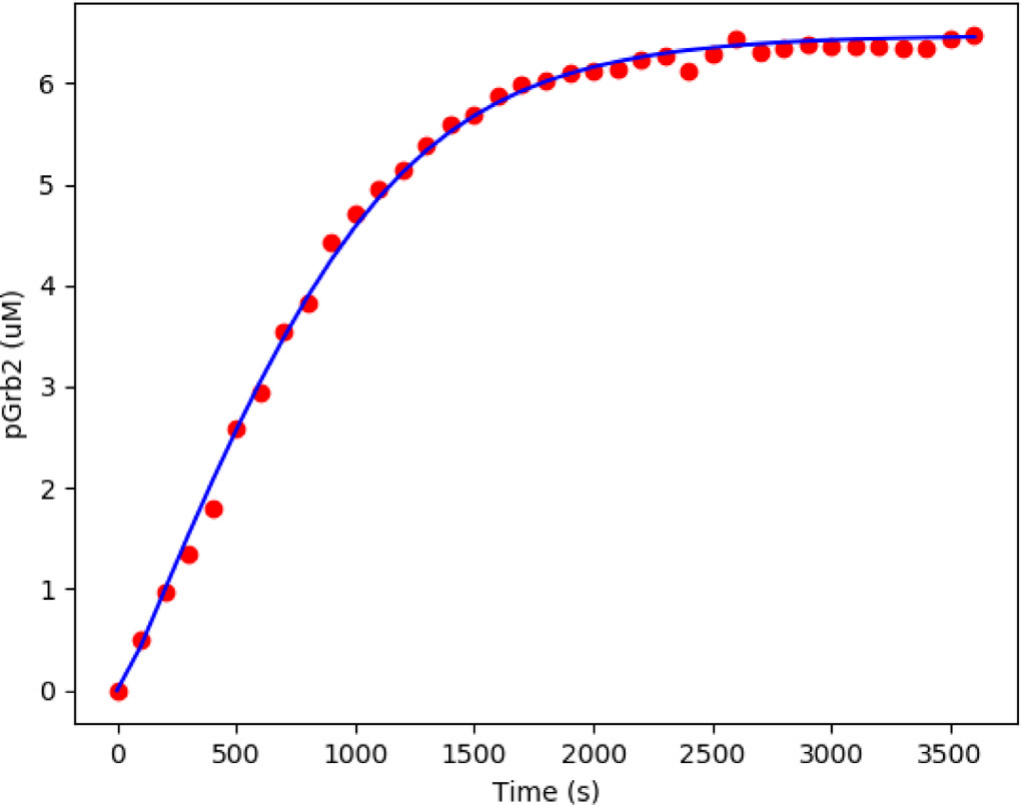
The best fit for phosphorylation of Grb2 with the experimental observations of Tsang et al. [23]. The simulation time was 3500 s. The RMSE for the model is 0.0695 μM. The model predictions represented the experimental data very well. Blue curves show model predictions and red points are observed experimental data in Tsang et al. [23]. The SED-ML associated with this model can be found at https://github.com/Nurulizza/FCepsilonRI/blob/master/FCepsilonRI_Tsangdata.sedml.

FCεRIγ appears as Level 2 models in Figure 2. The repository associated with this model can be found at https://github.com/Nurulizza/FCepsilonRI. The simulated Grb2 phosphorylation was a good fit with the experimental observations of Tsang et al. [23]. The model and observed experimental kinetics of phosphorylation of Grb2 are shown in Figure 9. The root mean square (RMS) error for the model is 0.0695 µM. The RMSE is 1.07% of the peak concentration. Visual inspection of the graph confirms this. The best fit to this data set was achieved with the parameters listed in Table 5.

**Table 5:** Fitted parameters to experimental data from Tsang et al. [23]. parameter estimation was performed with each unknown kinetic rate constant allowed to vary in a feasible range that is between 1×10^−3^ and 1×10^2^[9]. However, we expand the range when we see the optimisation error potentially decreasing with larger boundary.

Parameter	Description	Value	Source
k_f1_	FC-pLyn binding rate	58.3902 µM^−1^.s^−1^	Fitted value
k_f2_	FC phosphorylation rate	0.0082 s^−1^	Fitted value
k_f3_	Lyn phosphorylation rate	1.0887 µM^−1^.s^−1^	Fitted value
k_f4_	pFC-Syk binding rate	10.5797 µM^−1^.s^−1^	Fitted value
k_f5_	Syk phosphorylation rate	63.6727 s^−1^	Fitted value
k_f6_	pSyk-Grb2 binding rate	0.4143 µM^−1^.s^−1^	Fitted value
k_f7_	Grb2 phosphorylation rate	11.4185 s^−1^	Fitted value
k_r1_	FC-pLyn dissociation rate	0.0136 s^−1^	Fitted value
k_r4_	pFC-Syk dissociation rate	0.0807 s^−1^	Fitted value
k_r6_	pSyk-Grb2 dissociation rate	0.7313 s^−1^	Fitted value

Knowledge of which parameters the model is sensitive to helped to establish which parameters needed to be fixed when we tried to predict to another set of data (e.g., the phosphorylation of Grb2). Figures 1 and 2 in Additional File 1 presents the differences in pSyk solutions within the feasible parameter range for all rate constants in this model. We varied each parameter, one at a time, over the feasible parameter range to check how much they affected the solutions while keeping other rate constant parameters fixed.

For forward constants k_f2_, k_f4_, k_f6_ and k_f7_, we observed a minimum in the error corresponding to the fitted parameter value which showed the fitting had converged to something appropriate where most parameters were very close to at least a local minimum in the parameter space. It means the model is sensitive to those parameters. The sensitive parameters are the dissociation rate of phosphorylated FCεRIγ and Lyn complex, k_f2_, association of phosphorylated FCεRIγ and Syk, k_f4_, association of pSyk and Grb2, k_f6_, and dissociation of pSyk and Grb2 complex, k_f7_. These parameters remained fixed in subsequent simulations. Constant forward rate parameters k_f1_ and reverse rate parameters k_r1_, k_r4_ and k_r6_ showed distinct changes in model behaviour on one side of the minimum. In this case we can vary these parameters during fitting to another set of data in the range around the minimum in which the line is flat. A flat line was found for k_f1_ and P_i_ showing that the model is not sensitive to those parameters (data not shown). The latter two types of parameter could therefore be varied over the whole defined range in fitting to subsequent data sets.

Parameter fitting was then performed by fixing the parameters that the previous fitted model was sensitive to: we fixed the value of k_f2_, k_f4_ k_f6_ and k_f7_ (see Table 6). In this subsequent fitting, for constant forward rate parameters k_f1_ and reverse rate parameters k_r1_, k_r4_ and k_r6_ that showed distinct changes in model behaviour on one side of the minimum, we varied the parameters only over the range that model predictions for the Tsang data were not sensitive to these parameters [23]. The list of kinetic rate constants that we are going to fit and the boundaries are given in Table 7.

**Table 6:** Fixed kinetic parameters in this subsequence fitting. These parameters are kinetic rates where the model is sensitive to.

Parameter	Value	Units
k_f2_	0.0081	s^−1^
k_f4_	10.597	µM^−1^. s^−1^
k_f6_	0.4143	µM^−1^. s^−1^
k_f7_	11.4185	s^−1^

**Table 7:** Kinetic parameters to fit to data in Faeder et al. [24] and the boundaries condition. Parameters k_f1_, k_r1_, k_r4_ and k_r6_ showed distinct changes in model behaviour on one side of the minimum, so we varied the parameters only over the range that model predictions for the Tsang data were not sensitive to these parameters.

Parameter	Lower bound	Upper bound
k_f1_	10^−1.5^	10^2^
k_f3_	10^−3^	10^2^
k_f5_	10^1^	10^2^
k_r1_	10^−3^	10^1^
k_r4_	10^−3^	10^0.5^
k_r6_	10^−3^	10^0^
Pi	10^−3^	10^2^

**Fig. 10:**
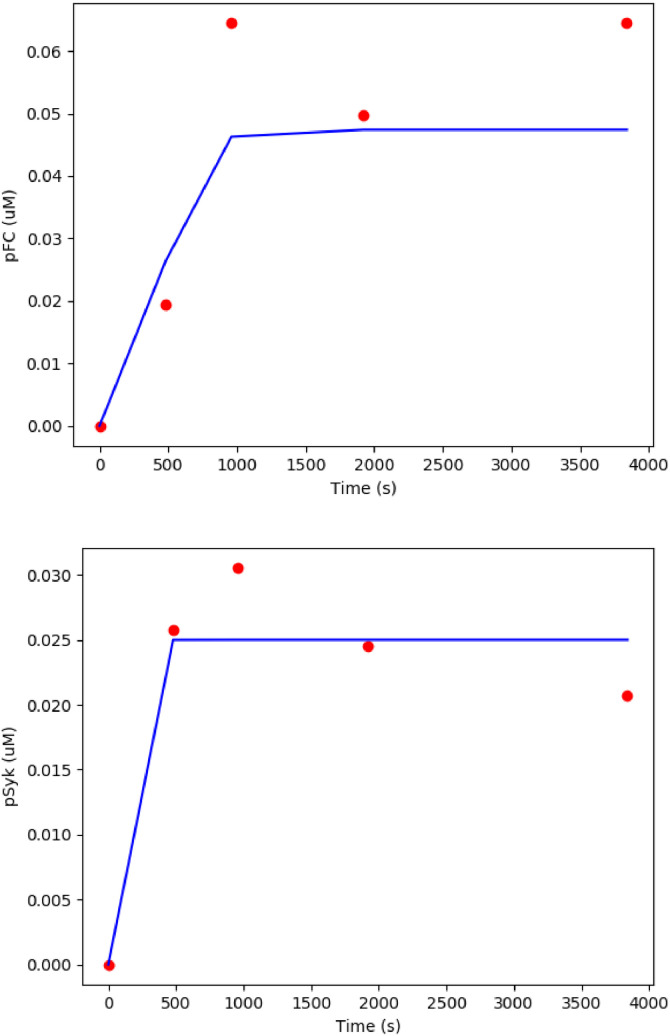
The best fit for phosphorylation of FCεRIγ (pFC) (top) and phosphorylation of Syk (bottom). The simulation time was 4000 s. The RMSEs are 0.0089 μM for FC phosphorylation and 0.0022 μM for Syk phosphorylation. These best fits are not perfect representations of the data, however, the available data are relatively sparse in time, and appear to contain more noise, so it may not be possible to obtain a clearly better fit to these data points. Blue curves show model predictions and red points are observed experimental data from Faeder et al. [24]. The SED-ML associated with this model is available at https://github.com/Nurulizza/FCepsilonRI/blob/master/FCepsilonRI_Faederdata.sedml.

The best fit was achieved with the parameters listed in Table 8. The model and the observed experimental [24] kinetics of phosphorylation of FCεRIγ and Syk are shown in Figure 10. The RMSEs are 0.0089 µM for FCεRIγ phosphorylation and 0.0022 µM for Syk phosphorylation. These RMS errors are approximately 10% of the peak values for pFCεRIγ and pSyk. Along with Figure 8 these best fits are not perfect representations of the data, however, the available data are relatively sparse in time, and appear to contain more noise, so it may not be possible to obtain a clearly better fit to these data points.

**Table 8:** Re-fitted parameters that the previous fitted model was not sensitive to. The model was fitted to data set from Faeder et al. [24]. The previous parameters is shown in Table 5.

Parameter	Description	Value	Source
k_f1_	FC-pLyn binding rate	54.7678 µM^−1^. s^−1^	Fitted value
k_f3_	Lyn phosphorylation rate	0.0035 µM^−1^. s^−1^	Fitted value
k_f5_	Syk phosphorylation rate	33.7157 s^−1^	Fitted value
k_r1_	FC-pLyn dissociation rate	0.0031 s^−1^	Fitted value
k_r4_	pFC-Syk dissociation rate	0.1174 s^−1^	Fitted value
k_r6_	pSyk-Grb2 dissociation	0.481 s^−1^	Fitted value

However, the model predictions shown in Figure 8 does not predict the experimentally observed drop in pSyk after 1000 s due to formation of pSyk-Grb2 complex. This may be because the concentration of Grb2 in the model was low (0.01 µM), which limited its impact on pSyk. The initial condition of Grb2 was fitted at 0.01 µM to get a good fit to the experimental data. The parameters for FCεRIγ model fitted to data set from Tsang et al. [23] and Faeder et al. [24] are listed in Table 9. The sensitivity analysis of the model is provided in Additional File 2.

**Table 9:** The parameters for FCεRIγ model fitted to data set from Tsang et al. [23] and Faeder et al. [24]. Status of each parameter that can be fitted or fixed in calibration of subsequent components due to the sensitivity analysis is listed in the table.

Parameter	Value	Source	Status
k_f1_	54.7678 µM^−1^.s^−1^	Fitted to [24]	Fit
k_f2_	0.0082 s^−1^	Fitted to [23]	Fix
k_f3_	0.0035 µM^−1^.s^−1^	Fitted to [24]	Fix
k_f4_	10.5797 µM^−1^.s^−1^	Fitted to [23]	Fit
k_f5_	33.7157 s^−1^	Fitted to [24]	Fit
k_f6_	0.4143 µM^−1^.s^−1^	Fitted to [23]	Fit
k_f7_	11.4185 s^−1^	Fitted to [23]	Fix
k_r1_	0.0031 s^−1^	Fitted to [24]	Fit
k_r4_	1.1174 s^−1^	Fitted to [24]	Fit
k_r6_	0.481 s^−1^	Fitted to [24]	Fit

The sensitivity analysis showed that the model fitted to the data measured by Faeder et al. [24] is sensitive to the dissociation rate of phosphorylated FCεRIγ and Lyn complex, k_f2_, the phosphorylation rate of Lyn, kf3 and Pi for the pFC. The same parameters especially k_f3_ and P_i_, however, do not contribute to pSyk variability. The model fitted the experimental data well with large values of parameters k_f1_, k_f4_ and k_f5_. Sensitivity analysis showed that the model converges toward minimum error for both FCεRIγ and Syk phosphorylation on the right region of the parameter space. In contrast, parameters k_r1_ gave the best fit with small values when the model converges toward minimum error for both FCεRIγ and Syk phosphorylation on the left region of the parameter space. The pathway can be calibrated around the regions within the minimum error of parameters space. The model was not sensitive to the dissociation rate of pFCεRIγ and Syk, k_r4_, association of pSyk and Grb2, k_f6_, dissociation of pSyk and Grb2, k_r6_ and phosphorylation of Grb2, k_f7_ (data not shown). Those parameters are therefore free to be varied in calibration of subsequent components of the model that incorporate this pathway.

Lyn kinase phosphorylates FCεRIγ into phosphorylated FCεRIγ. Re-phosphorylation of Lyn then occurs. Due to a lack of quantitative information about the aggregation rate of FCεRIγ in the literature, we constructed a simple model of the system, in which we assumed that the FCεRIγ was in the aggregation state. The model neglects aspects of the complex dynamics of the FCεRIγ system, which has previously been incorporated into mathematical descriptions. This means, that although it captures the dynamics of the available experimental data adequately, it may be shown in the future to neglect important dynamics, as more experimental data becomes available. The modular structure of this modelling approach would allow the component of the model to be replaced with a more detailed morel in the future, if necessary.

Phosphorylated FCεRIγ activates Syk through binding activity. The activation of Syk eliminates the lag phase in Syk activity and results in a linear rate of Grb2 phosphorylation, indicating that FCεRIγ binding activates Syk. Observations using the model displayed qualitatively similar pFCεRIγ-Syk binding behaviour to the available experimental data.

Experimental work by Tsang et al. [24] showed that Syk is an OR-gate switch, meaning that it can reach full activation either by ITAM binding or autophosphorylation activation. Tsang et al. demonstrated that activation by both stimuli works through the same mechanism and the application of both stimuli is expected to give only a small increase in activity [23]. Our model supports this and implies that ITAM binding is sufficient to cause full activation of Syk. Phosphorylated Syk sustains activity over time to facilitate longer-term changes in cell signalling.

In our model, the Syk is an OR-gate switch, meaning that it can reach full activation with one factor by ITAM binding. The ability of Syk to reach full activation with a single stimulus helps to define the ability of Syk to sustain its activity over time although after transient activation of ITAM to facilitate longer-term changes in cell signalling. As an example, Syk activity is required for more than 1 hour to induce activation of NFAT transcription [23].

Phosphorylation of Grb2 is dependent on the available concentration of Syk and Grb2 in the cell. The simulation results showed that the model could produce quantitatively similar kinetic behaviour in the phosphorylation of Grb2 to the experimental data. Phosphorylation of Grb2 was initially linear with time and then plateaued after all the Grb2 was completely phosphorylated. The lag in the reaction time was eliminated.

In conclusion, a minimal FCεRIγ model was developed and implemented in CellML. The results for phosphorylation of Grb2 for a data set from Tsang et al. [23] were presented to validate and test for phosphorylation of FCεRIγ and Syk in a data set from Faeder et al. [24]. A basic sensitivity analysis was performed to study the effect of the model constants’ parameters on the model.

#### HLA-G cytokines model

**Fig. 11:**
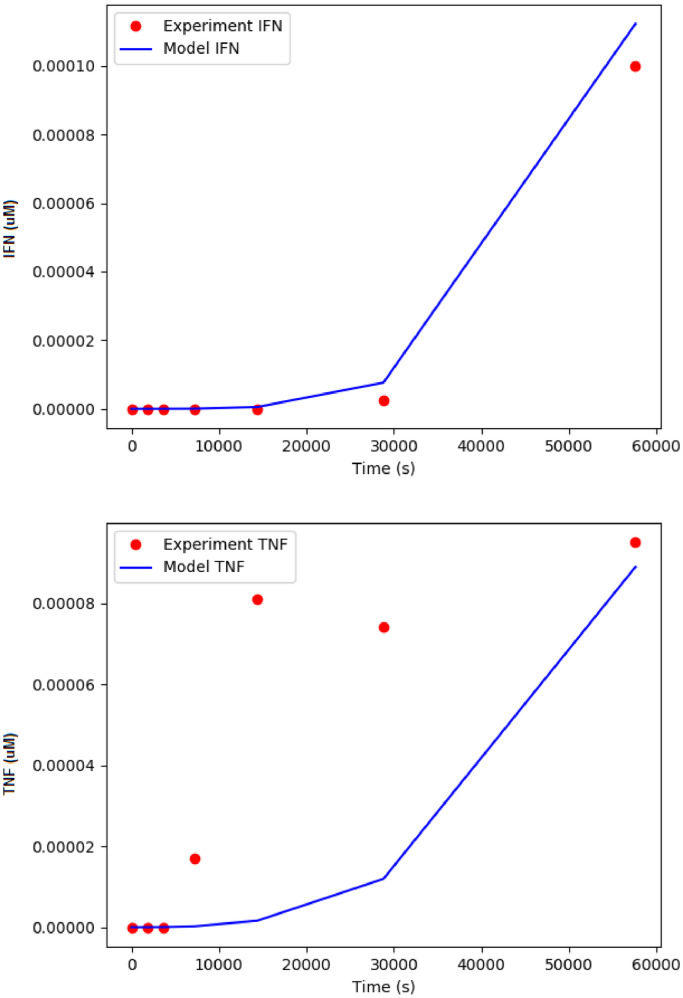
Fitted model to data from Rajagopalan et al. [26] of (a) IFNγ and (b) TNFα intracellular cytokine production. The simulation time was 60000 s. The model prediction represented IFNγ cytokine secretion experimental data well, however the model did not represented TNFα secretion very well. Blue curves show model predictions and red points are observed experimental data from Rajagopalan et al. [23]. The SED-ML associated with this model is available at https://github.com/Nurulizza/HLAG_to_cytokine/blob/master/dl4cytokines.sedml.

This section introduces the model labelled HLA-G cytokines in Figure 2. The repository associated with this model can be found at https://github.com/Nurulizza/HLAG_to_cytokine. The fit of the model to the experimental data shown in Figure 11. The simulated cytokine output showed similar behaviour for IFNγ secretion as the experiment. The model did not fit as well to TNFα secretion as shown in the figure. With the limitation of knowledge and data on the pathway inside the nucleus, we simplified the pathway by assuming the cytokine secretion is proportional to the NFAT translocation into the nucleus. In the model, the production of IFNγ and TNFα are k_f4_ and k_f5_.

In the study by Rajagopalan et al. [26], the expression of genes expression was confirmed by using RT-PCR analysis. They observed time course differences in the kinetic responses. TNFα was detected within the first two hours, whilst there is delayed secretion of IFNγ compared to TNFα and other cytokines in the study. The delay in IFNγ release was confirmed by RT-PCR analysis where induction of IFNG transcription happened only 8 hours after stimulation. IFNG gene transcription requires de novo protein synthesis since it can be inhibited by cycloheximide that caused late response (Y.T. Bryceson, unpublished observation) [26].

The sensitivity analysis shows that IFNγ and TNFα productions are sensitive to both parameters, meaning that the changes of any parameter will affect the secretion of both cytokines. To elevate the secretion of TNFα at 8,000 s causes bad fit to IFNγ secretions. In this multi-objective optimisation, the model was only able to represent the IFNγ data accurately. When more data become available in the future, especially the knowledge of signalling pathway inside the nucleus, we hope the fitting can be improved. The model, however, captured the delay in cytokine production as expected. The best fit achieved with the parameters listed in Table 10.

**Table 10:** Fitted parameters to data from Rajagopalan et al. [26]. The description of each parameters is provided in column 2.

Parameter	Description	Value	Source
hGactivating/k_f10_	KIR2DL4-HLA-G binding rate	0.0141 µM^−1^.s^−1^	Fitted value
hGactivating/k_r10_	KIR2DL4-HLA-G dissociation rate	0.0140 s^−1^	Fitted value
FCepsilonRI/k_f1_	pLyn binding rate	6.1804 µM^−1^.s^−1^	Fitted value
FCepsilonRI/k_f5_	Syk phosphorylation rate	0.8040 s^−1^	Fitted value
FCepsilonRI/k_r1_	pLyn dissociation rate	0.0015 s^−1^	Fitted value
FCepsilonRI/k_r4_	Syk dissociation rate	0.13486 s^−1^	Fitted value
FCepsilonRI/k_r6_	Grb2 dissociation rate	0.71853 s^−1^	Fitted value
hG_FC/k_f21_	FC binding rate	0.0165 µM^−1^.s^−1^	Fitted value
hG_FC/k_r21_	FC dissociation rate	0.0517 s^−1^	Fitted value
PI3K/k_f2_	pGrb2-PI3K binding rate	8.9165 µM^−1^.s^−1^	Fitted value
PI3K/k_r2_	pGrb2-PI3K dissociation rate	0.0061 s^−1^	Fitted value
PI3K/k_f3_	PI3K activation rate	14.6231 s^−1^	Fitted value
dupont_NFAT/k_f_	IP3-NFAT binding rate	0.0065 s^−1^	Fitted value
NFAT_cytokine/k_f4_	IFNγ production rate	0.0684 s^−1^	Fitted value
NFAT_cytokine/k_f5_	TNFα production rate	23.1163 s^−1^	Fitted value
NFAT_cytokine/kf_31_	TNFα secretion rate	0.0291 s^−1^	Fitted value
NFAT_cytokine/kf_32_	IFNγ secretion rate	9.7858e-03 s^−1^	Fitted value
NFAT Cycling equation/kf_21_	NFATp_c_ binding rate	0.0516 µM^−1^.s^−1^	Fitted value
NFAT Cycling equation/kf_22_	NFATN_c_ translocation rate	0.0030 s^−1^	Fitted value
NFAT Cycling equation/kf_23_	NFATNn rephosphorylation rate	0.0022 s^−1^	Fitted value
NFAT Cycling equation/kf_24_	NFATNp_n_ translocation rate	0.9844 s^−1^	Fitted value
NFAT Cycling equations/k_r21_	NFATp_c_ dissociation rate	2.0772 s^−1^	Fitted value
NFAT Cycling equations/k_r23_	NFATp_n_ rephosphorylation rate	0.3345 µM^−1^.s^−1^	Fitted value

The sensitivity analysis of the model is provided in Additional File 3. The sensitivity analysis of the model shows that the model is sensitive to parameters k_f2_, k_f21_, k_f22_, k_f23_ and k_r21_. The model is also sensitive to parameters k_f4_, k_f5_ and k_f31_ in component cytokines. The analysis showed that parameters k_f21_ depicts the binding activated CaN to NFATp_c_ to form the complex NFATN_c_, k_f21_ and the nuclear import rate, k_f22_ are the most sensitive parameters that gave big difference to the model within the tested parameter space, that are between 10^0^ and 10^2^ and between 10^−2^ and 10^2^. Parameters k_f23_ and k_f2_ sit on minimum within the edge of regions within the tested parameters space. Parameters k_f5_ and k_f31_ are significant to either the production of IFNγ or TNFα.

We further sample parameter space with an aim to cover the range of feasible parameter combinations without the requirement for an unfeasibly large number of model runs. Again, we achieve this by using the inbuilt Saltelli sampling function in the python SALib library. The mean and standard deviation for the experimental data is reported in Table 11. After we ran the script until the end of 4800 simulations, 302 alternate solutions were found that could give different predictions of the model behaviour. The ssq of the alternate solutions range from 3.16e-9 to 1.90-8. This means that over the range of parameter space assessed, the ’best fit’ solution obtained from parameter fitting was not unique. It gives some confidence that the model provides many satisfactory solutions. The list of the alternate solutions is provided at https://github.com/Nurulizza/HLAG_to_cytokine/tree/master/Alternative_solutions. A list of ssq for the solutions is provided in Additional File 4.

**Table 11:** The mean and standard deviation use for uncertainty quantification.

Cytokine	Mean (uM)	Standard deviation (uM)
TNFα	9.5e-5	9.5e-5
IFNγ	1.0e-4	1.0e-4

## Declaration of Competing Interests

The authors declare that they have no known competing financial interests or personal relationships that could have appeared to influence the work reported in this paper.

## Data Availability

I have shared the link to my data/code.

## Appendix Supplementary materials

Image, application 1

Image, application 2

Image, application 3

Image, application 4
