# Supplementary material for: Modularisation of published and novel models toward a complex KIR2DL4 pathway in pbNK cell
Source: MethodsX. 2022 Jun 16;9:101760. doi: 10.1016/j.mex.2022.101760 (PMC9237949; doi:10.1016/j.mex.2022.101760)
Supplement: Supplementary file 3 [file mmc3.pdf]

Additional File 3

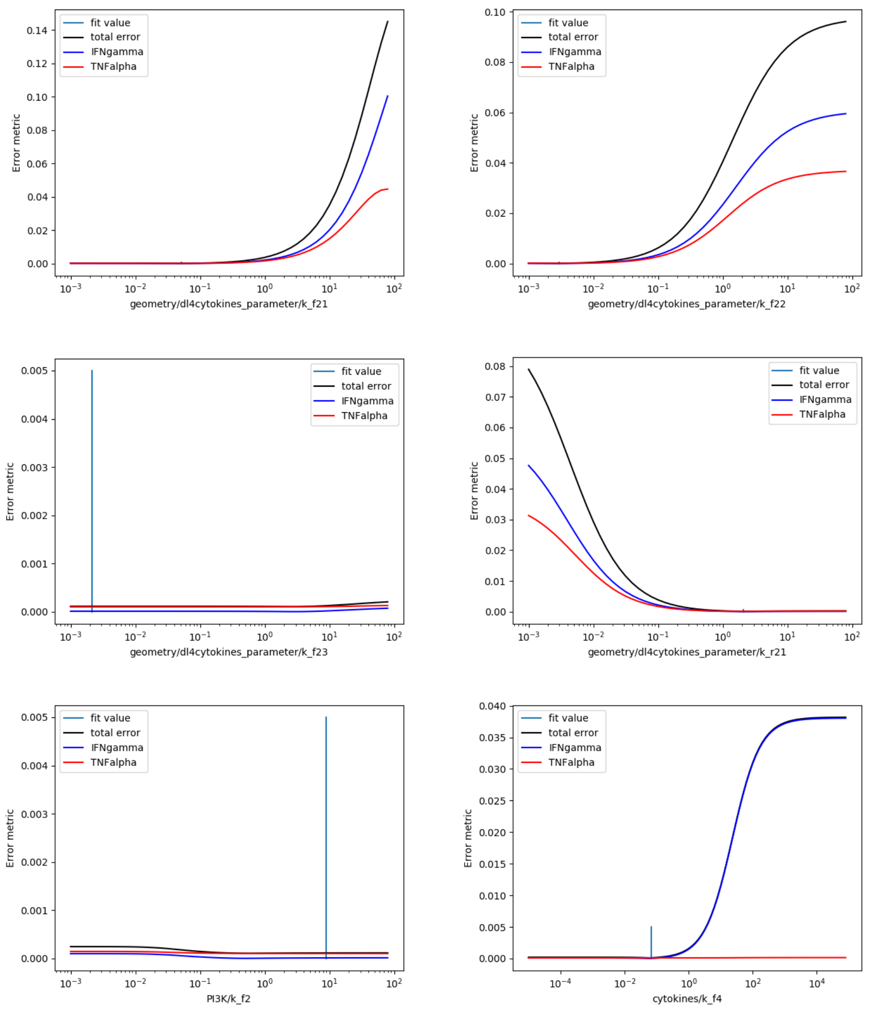

Figure 1: The sensitivity of the HLA-G cytokines model solutions around the 'best fit' to a selection of parameters. The model was initialised with the best fit values for each parameter (fit value), and run multiple times over the parameter range we assumed it to take (as shown on the x-axis). Error is shown in red. These results are continued in Figure 2.

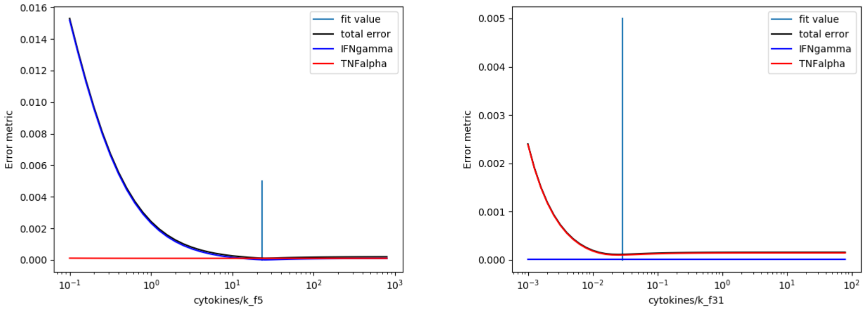

Figure 2: Continuing the results begun in Figure 1 with the sensitivity of model solutions around the 'best fit' to a selection of parameters. The sensitivity of the HLA-G cytokines model solutions around the 'best fit' to a selection of parameters. The model was initialised with the best fit values for each parameter (fit value), and run multiple times over the parameter range we assumed it to take (as shown on the x-axis). Error is shown in red.
