# Supplementary material for: Modularisation of published and novel models toward a complex KIR2DL4 pathway in pbNK cell
Source: MethodsX. 2022 Jun 16;9:101760. doi: 10.1016/j.mex.2022.101760 (PMC9237949; doi:10.1016/j.mex.2022.101760)
Supplement: Supplementary file 4 [file mmc4.pdf]

Solution	ssq
0	1.50E-08
1	1.76E-08
2	7.76E-09
3	1.86E-08
4	1.90E-08
5	1.87E-08
6	1.87E-08
7	9.16E-09
8	1.21E-08
9	9.08E-09
10	1.90E-08
11	1.90E-08
12	1.90E-08
13	1.90E-08
14	1.90E-08
15	1.90E-08
16	1.90E-08
17	1.90E-08
18	1.90E-08
19	1.90E-08
20	1.90E-08
21	1.89E-08
22	1.88E-08
23	1.90E-08
24	1.90E-08
25	1.90E-08
26	1.90E-08
27	1.90E-08
28	1.90E-08
29	1.90E-08
30	1.90E-08
31	1.90E-08
32	1.80E-08
33	1.90E-08
34	1.85E-08
35	1.90E-08
36	1.75E-08
37	1.82E-08
38	1.81E-08
39	1.83E-08
40	1.73E-08
41	1.75E-08
42	3.63E-09
43	1.78E-08
44	1.82E-08
45	1.82E-08
46	1.83E-08

Solution	ssq
47	1.82E-08
48	1.03E-08
49	1.82E-08
50	1.82E-08
51	1.82E-08
52	5.46E-09
53	1.82E-08
54	9.06E-09
55	9.06E-09
56	9.06E-09
57	1.80E-08
58	9.06E-09
59	9.06E-09
60	9.06E-09
61	9.06E-09
62	9.06E-09
63	9.06E-09
64	1.85E-08
65	1.88E-08
66	7.93E-09
67	1.59E-08
68	9.30E-09
69	9.30E-09
70	9.30E-09
71	9.30E-09
72	9.30E-09
73	9.30E-09
74	9.16E-09
75	1.90E-08
76	1.90E-08
77	9.08E-09
78	9.26E-09
79	5.02E-09
80	9.12E-09
81	1.70E-08
82	8.87E-09
83	1.90E-08
84	1.25E-08
85	9.15E-09
86	1.87E-08
87	1.88E-08
88	9.07E-09
89	9.33E-09
90	9.07E-09
91	1.35E-08
92	9.07E-09
93	9.07E-09

Solution	ssq
94	9.07E-09
95	1.27E-08
96	9.07E-09
97	9.81E-09
98	9.83E-09
99	1.37E-08
100	1.65E-08
101	1.89E-08
102	1.89E-08
103	1.45E-08
104	1.89E-08
105	1.89E-08
106	1.63E-08
107	1.89E-08
108	1.89E-08
109	1.90E-08
110	1.89E-08
111	1.89E-08
112	1.89E-08
113	1.89E-08
114	1.89E-08
115	1.89E-08
116	1.90E-08
117	1.89E-08
118	1.89E-08
119	1.89E-08
120	1.90E-08
121	1.90E-08
122	1.90E-08
123	1.63E-08
124	1.87E-08
125	1.64E-08
126	1.88E-08
127	1.63E-08
128	1.01E-08
129	1.65E-08
130	1.62E-08
131	1.61E-08
132	1.90E-08
133	1.85E-08
134	1.80E-08
135	1.63E-08
136	1.63E-08
137	1.85E-08
138	1.63E-08
139	1.76E-08

Solution	ssq
140	1.63E-08
141	1.63E-08
142	1.63E-08
143	1.54E-08
144	1.88E-08
145	1.88E-08
146	1.90E-08
147	1.90E-08
148	9.13E-09
149	9.12E-09
150	1.90E-08
151	1.90E-08
152	1.41E-08
153	1.89E-08
154	1.90E-08
155	1.90E-08
156	1.90E-08
157	1.86E-08
158	1.89E-08
159	1.90E-08
160	1.90E-08
161	1.90E-08
162	1.90E-08
163	1.90E-08
164	1.90E-08
165	1.90E-08
166	1.90E-08
167	1.90E-08
168	1.90E-08
169	1.90E-08
170	1.90E-08
171	1.90E-08
172	1.90E-08
173	1.90E-08
174	1.90E-08
175	1.90E-08
176	1.90E-08
177	1.90E-08
178	1.90E-08
179	1.90E-08
180	1.90E-08
181	1.90E-08
182	1.90E-08
183	1.90E-08
184	1.90E-08
185	1.90E-08

Solution	ssq
186	1.90E-08
187	1.90E-08
188	1.90E-08
189	1.90E-08
190	1.90E-08
191	1.90E-08
192	1.00E-08
193	1.25E-08
194	1.90E-08
195	1.90E-08
196	1.90E-08
197	1.90E-08
198	1.90E-08
199	1.90E-08
200	1.90E-08
201	1.90E-08
202	1.90E-08
203	1.90E-08
204	1.90E-08
205	1.90E-08
206	1.90E-08
207	1.90E-08
208	1.90E-08
209	1.90E-08
210	1.90E-08
211	1.90E-08
212	1.90E-08
213	1.90E-08
214	1.90E-08
215	1.90E-08
216	1.90E-08
217	1.75E-08
218	1.11E-08
219	1.11E-08
220	1.74E-08
221	1.12E-08
222	1.11E-08
223	1.09E-08
224	1.11E-08
225	1.09E-08
226	1.10E-08
227	1.15E-08
228	1.11E-08
229	1.11E-08
230	9.28E-09

Solution	ssq
231	1.11E-08
232	1.42E-08
233	1.11E-08
234	1.11E-08
235	1.11E-08
236	9.75E-09
237	1.10E-08
238	1.88E-08
239	4.29E-09
240	9.20E-09
241	1.64E-08
242	1.75E-08
243	1.56E-08
244	1.89E-08
245	1.84E-08
246	1.78E-08
247	1.22E-08
248	1.64E-08
249	1.79E-08
250	1.75E-08
251	1.63E-08
252	1.62E-08
253	1.89E-08
254	1.34E-08
255	1.62E-08
256	1.68E-08
257	1.75E-08
258	1.63E-08
259	1.62E-08
260	1.62E-08
261	1.62E-08
262	1.62E-08
263	1.61E-08
264	1.62E-08
265	1.63E-08
266	1.62E-08
267	1.82E-08
268	1.62E-08
269	1.62E-08
270	1.42E-08
271	3.16E-09
272	1.42E-08
273	1.42E-08
274	1.19E-08
275	1.90E-08

Solution	ssq
276	1.42E-08
277	1.44E-08
278	1.90E-08
279	1.79E-08
280	1.42E-08
281	1.42E-08
282	1.42E-08
283	1.42E-08
284	9.36E-09
285	1.42E-08
286	1.42E-08
287	1.42E-08
288	1.42E-08
289	1.10E-08
290	1.22E-08
291	1.09E-08
292	1.74E-08
293	1.11E-08
294	1.56E-08
295	1.25E-08
296	1.75E-08
297	1.60E-08
298	1.74E-08
299	1.75E-08
300	1.75E-08
301	1.75E-08
302	1.59E-08
